# Diminished viability of human ovarian cancer cells by antigen-specific delivery of carbon monoxide with a family of photoactivatable antibody-photoCORM conjugates†
†Electronic supplementary information (ESI) available: Synthetic scheme and calculations for CO release (Schemes S1 and S2). Spectroscopic, chromatography and mass spectrometry data (Fig. S1–S4, S8 and S9). Myoglobin assays and cell toxicity/viability data (Fig. S5–S7 and S10–S13). Description of the experimental procedures. See DOI: 10.1039/c9sc03166a


**DOI:** 10.1039/c9sc03166a

**Published:** 2019-11-20

**Authors:** Brian Kawahara, Lucy Gao, Whitaker Cohn, Julian P. Whitelegge, Suvajit Sen, Carla Janzen, Pradip K. Mascharak

**Affiliations:** a Department of Chemistry and Biochemistry , University of California , Santa Cruz , CA 95064 , USA . Email: pradip@ucsc.edu; b Pasarow Mass Spectrometry Laboratory , Jane and Terry Semel Institute for Neuroscience and Human Behavior , University of California at Los Angeles , Los Angeles , CA 90095 , USA; c Department of Obstetrics and Gynecology , David Geffen School of Medicine , University of California at Los Angeles , Los Angeles , CA 90095 , USA

## Abstract

Carbon monoxide (CO)-releasing antibody conjugates were synthesized utilizing a photoactivatable CO-releasing molecule (photoCORM) and mouse monoclonal antibodies linked by a biotin-streptavidin system. Different monoclonal antibodies raised against different surface-expressed antigens that are implicated in ovarian cancer afforded a family of antibody-photoCORM conjugates (Ab-photoCORMs). In an immunosorbent/cell viability assay, Ab-photoCORMs accumulated onto ovarian cancer cells expressing the target antigens, delivering cytotoxic doses of CO *in vitro*. The results described here provide the first example of an “immunoCORM”, a proof-of-the-concept antibody-drug conjugate that delivers a gaseous molecule as a warhead to ovarian cancer.

## Abstract 2

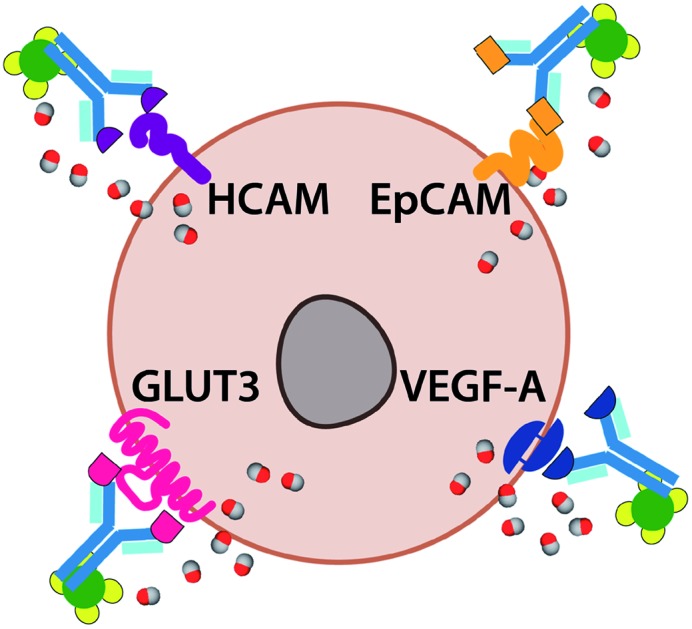
Antibodies conjugated to a photoactive transition metal carbonyl complex afford antigen-directed delivery of cytotoxic carbon monoxide to ovarian cancer cells.

## Introduction

Carbon monoxide (CO), while long recognized as a toxic gas, is an endogenously produced gasotransmitter that regulates immune/inflammatory processes and vascular tone through reactions with heme-containing proteins.^1^ In recent years, exogenous delivery of CO has shown promise as a novel therapeutic in a variety of disease and injury models, including cancer.^2^

The challenge of delivering efficacious concentrations of CO to a target tissue has been approached by our group and others by synthesizing CO-releasing molecules (CORMs) with properties necessary for a potential therapeutic, including water solubility,^3^ incorporation within biocompatible materials^4^,^5^ and controllable release of CO.^6^–^9^ Very recently, with the use of photoactivatable CORMs (photoCORMs) our group has elucidated mechanism(s) by which CO exerts deleterious effects against human breast and ovarian cancer cell models.^10^,^11^ In such studies we have observed sensitization of ovarian cancer cells to drugs like cisplatin and paclitaxel through co-administration of CO.^11^ Because sensitization to conventional chemotherapeutics could mitigate the poor outcome of ovarian cancer treatment, precise target-specific delivery of CO to the malignant tissue appears to be a very desirable goal.

Although a number of CORMs and photoCORMs has been developed in recent years,^1^,^2^,^6^–^9^ most have notably lacked the ability to highly discriminate between targeted *versus* non-targeted tissues. With this in mind, we sought to conjugate a photoCORM to a monoclonal antibody with the goal of improving target specificity of CO-release. Antibody-drug conjugates (ADCs) are fast emerging as an effective strategy for anticancer therapies. In most cases small molecule drugs are combined with monoclonal antibodies to achieve high selectivity.^12^ Conjugation of photoCORMs (*i.e.* the warhead) to monoclonal antibodies using a biotin-streptavidin linker is a novel, currently unexplored and potentially effective strategy that could be employed for the controlled delivery of CO to specific tissues.

Herein we report the successful conjugation of a biotinylated-photoCORM to streptavidin-conjugated mouse monoclonal immunoglobulin G (IgG) antibodies to isolate Ab-photoCORMs for the controlled delivery of CO to ovarian cancer cell cultures with high specificity. Utilizing different monoclonal antibodies, a family of Ab-photoCORMs was synthesized with the goal of localizing and delivering cytotoxic levels of CO to ovarian cancer cells expressing different tumor-specific surface antigens. To the best of our knowledge, this communication is the first report of an antibody-drug conjugate in which the drug is a gaseous molecule, namely CO.

## Results and discussion

### Synthesis of biotinylated photoCORM (Complex **1**)

**Fig. 1:**
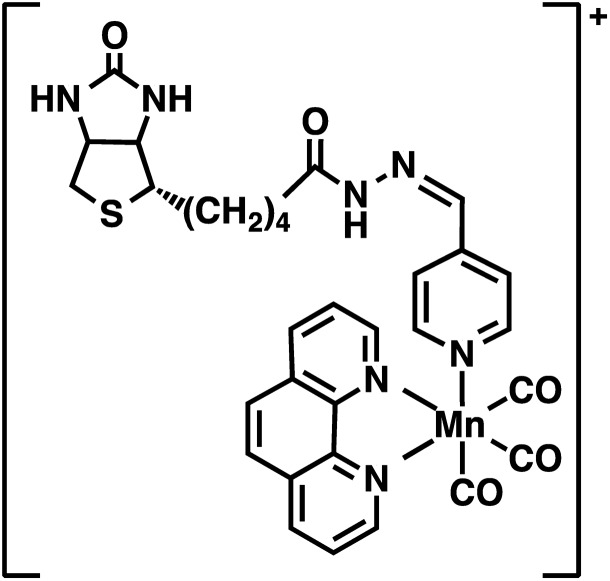
Structure of biotinylated photoCORM (Complex **1**).

The present work utilized a designed photoCORM [Mn(CO)_3_(phen)(4-pyAl)](CF_3_SO_3_) (where phen = 1,10-phenanthroline, 4-pyAl = pyridine-4-carboxaldehyde) as the photoactivatable CO donor. Biotinylation of this photoCORM (Fig. 1, Complex **1**) was achieved through reaction with biotin-hydrazide in trifluoroethanol at room temperature (Scheme S1†). The composition of Complex **1** was confirmed by electrospray ionization Fourier Transform mass spectrometry (ESI FTMS); (M^+^) *m*/*z* = 666.13539 (calculated for C_31_H_29_N_7_O_5_SMn: 666.13313, *Δ* ppm = 3.4 ppm, *Δ* mDa = 2.2) (Fig. S1†), and ^1^H NMR spectrum (ESI†). The infrared spectrum of Complex **1** showed the presence of two *ν*_C=O_ bands at 2039 and 1939 cm^–1^, characteristic of the manganese tricarbonyl moiety, and one *ν*_C=O_ band at 1685 cm^–1^ derived from the biotin unit (Fig. S2†). Electronic absorption spectra of solutions of Complex **1** in 1x phosphate-buffered saline (PBS) exhibited a broad absorbance band in the visible region between 320 and 450 nm (Fig. S3†). Exposure of Complex **1** to visible light resulted in systematic changes in the absorption spectra (Fig. S4†) arising from the loss of CO.^3^ Integration of the rate law for the photodegradation of Complex **1** was performed to determine pseudo-first order kinetics for CO release, with apparent visible light activated CO release rate *k*_app_ = 0.0030 ± 0.010 s^–1^ determined in 1x PBS (Fig. S4†). Complex **1** was stable in 1x PBS in the dark for ∼48 h, releasing CO only upon illumination with low power (10 mW cm^–2^), broadband, visible light (Fig. S5†). Furthermore, Complex **1** exhibited stability in human serum for 24 h at 37 °C, retaining the property of photorelease of CO, as confirmed by myoglobin assay (Fig. S6†).

Previous studies from this laboratory have demonstrated that sufficient levels of CO, delivered from photoCORMs, can induce apoptotic cell death in a wide variety of cancer cells.^4^,^5^,^13^–^15^ Likewise, Complex **1** upon illumination with visible light, significantly reduced cell viability in two ovarian cancer cell lines OVCAR-5 and SKOV-3 (ED_50_ = 48 and 25 µM respectively) assayed 24 h post-treatment (Fig. S7†).

### Synthesis of streptavidin-conjugated IgG (Complex **2**)

**Fig. 2:**
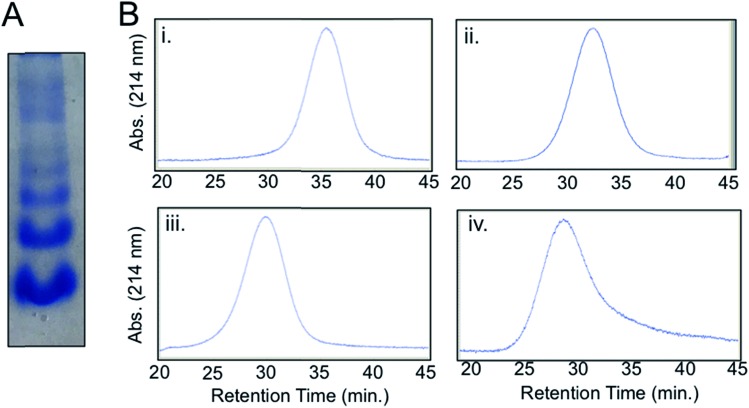
Complex **2**: streptavidin-conjugated IgG. (A) Native protein gel electrophoresis of crude Complex **2**. (B) Size-exclusion chromatograms of fractions of Complex **2**. [Retention time, ∼molecular weight, identity] (i) [35.4 min, ∼210 kDa, IgG + 1 streptavidin]. (ii) [32.2 min, ∼260 kDa, IgG + 2 streptavidin]. (iii) [29.9 min, ∼313 kDa, IgG + 3 streptavidin]. (iv) [28.6 min, ∼366 kDa, IgG + 4 streptavidin].

A streptavidin-biotin strategy was used to link Complex **1** to IgG, exploiting the strong affinity (*K*_d_ = 10^–14^M) and stability of the streptavidin-biotin interaction.^16^ The streptavidin-IgG conjugate was synthesized using a commercially available kit (ESI†). Native gel electrophoresis (Fig. 2A) and size exclusion chromatography (Fig. S8†) revealed conjugation of a variable number of streptavidin molecules to IgG which was expected as per manufacturer's notes. Fractionation of crude streptavidin-IgG conjugates following size exclusion chromatography was performed to resolve and isolate antibodies conjugated with 1–4 streptavidin molecules (Fig. 2B). These fractions were then pooled together (abbreviated hereafter as Complex **2**) for cellular studies.

### Construction of Ab-photoCORM conjugate

**Fig. 3:**
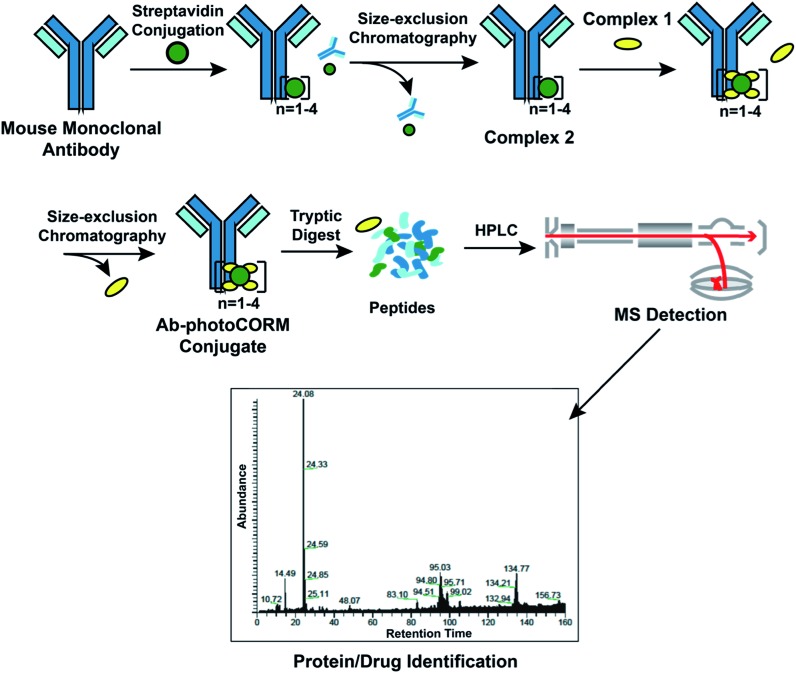
Synthesis and characterization of the antibody-photoCORM conjugate (Ab-photoCORM) and proteomic analysis of Ab-photoCORM. The scheme of bottom-up proteomics of the Ab-photoCORM is also shown.

Reaction of Complex **2** with excess Complex **1** afforded the antibody-photoCORM conjugate (Ab-photoCORM) through a streptavidin-biotin interaction (Fig. 3) (ESI†). The Ab-photoCORM was then purified to remove any trace of unbound streptavidin, unconjugated IgG and unincorporated Complex **1** by size-exclusion chromatography (Fig. 3).

**Fig. 4:**
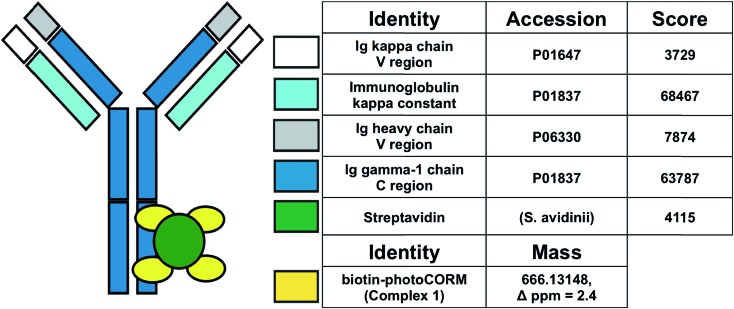
Proteomic scores of the Ab-photoCORMs synthesized in this study. Biotin-photoCORM (Complex **1**) was observed in the full MS scan of the tryptic digest of Ab-photoCORM. Protein scores greater than 67 are significant (*i.e. p* < 0.05).

Bottom-up proteomic analysis of the Ab-photoCORM confirmed the presence of streptavidin in the Ab-photoCORM (Fig. 4). Additionally, Complex **1** (M^+^) incorporated into the Ab-photoCORM was observed in the full MS scan (Fig. 4 and S9†). The Ab-photoCORM, by merit of Complex **1** incorporation, exhibited photo-activated release of CO, as determined by myoglobin assay performed in 1x PBS (Fig. S10†). Furthermore, the Ab-photoCORM exhibited stability in a biological fluid, as evidenced by its retained photo-activatable release of CO following incubation in human blood serum for 1 h at 37 °C (Fig. S11†).

**Fig. 5:**
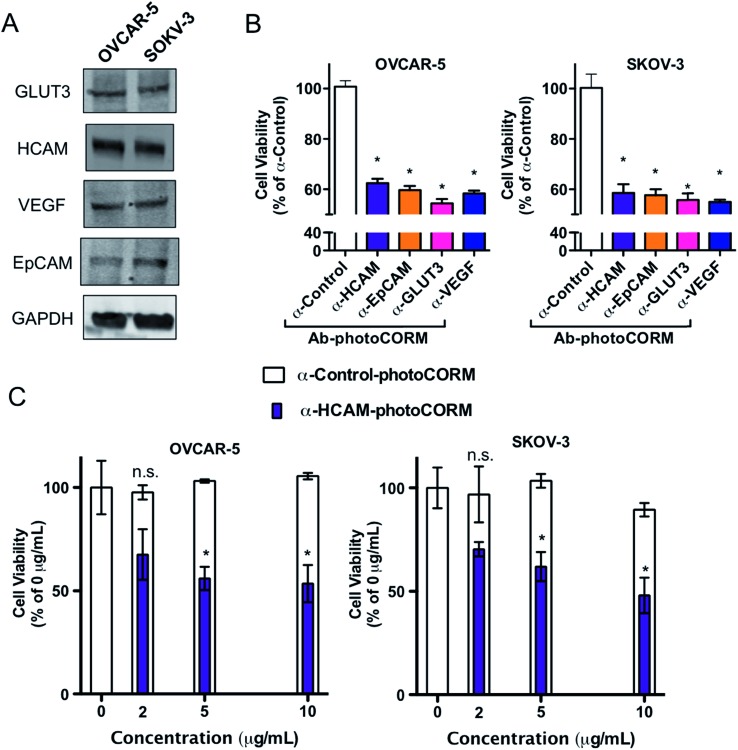
Antibody-photoCORM conjugates (Ab-photoCORMs) deliver cytotoxic levels to ovarian cancer cell lines *via* immunosorbent assay. (A) Western analysis of whole cell lysates of cell lines OVCAR-5 and SKOV-3, probing for antigens recognized by a family of Ab-photoCORMs. (B) Cell viability, as measured by cellular reduction of MTT, of OVCAR-5 and SKOV-3 24 h post-immunosorbent assay utilizing 2 µg mL^–1^ Ab-photoCORM conjugates. (C) Dose-dependency of α-HCAM-photoCORM, compared to α-Control-photoCORM, on cell viability. Data representative of *n* = 3 independent experiments. (*p < 0.05).

A family of Ab-photoCORM conjugates was synthesized (Table 1) using this synthetic strategy with commercially available mouse monoclonal IgG raised against four surface-expressed antigens implicated in ovarian cancer, namely homing cell adhesion molecule (HCAM),^17^ epithelial cell adhesion molecule (EpCAM),^18^ glucose transporter 3 (GLUT3),^19^ and vascular endothelial growth factor A (VEGF).^20^ Immunoblot analysis of whole cell lysates of cell line models utilized, OVCAR-5 and SKOV-3, confirmed the presence of the antigens recognized by the family of Ab-photoCORMs (Fig. 5A). An Ab-photoCORM utilizing IgG *not raised against any specific antigen* (α-Control-photoCORM) was also synthesized for application in cell viability experiments in order to account for any non-CO-mediated effects of the antigen-specific Ab-photoCORMs.

**Table 1:** Family of antibody-photoCORM conjugates (Ab-photoCORMs) synthesized from commercial antibodies, recognizing indicated human cell surface antigens implicated in ovarian cancer

Original mouse IgG	Epitote recognized	Streptavidin-IgG (Complex **2**)	Antibody-photoCORM conjugate (Ab-photoCORM)
HCAM (sc-7297)	Homing cell adhesion molecule (human)	Complex **2**-(α-HCAM)	α-HCAM-photoCORM
EpCAM (sc-53277)	Epithelial cell adhesion molecule (human)	Complex **2**-(α-EpCAM)	α-EpCAM-photoCORM
GLUT3 (sc-74399)	Glucose transporter 3 (human)	Complex **2**-(α-GLUT3)	α-GLUT3-photoCORM
VEGF-A (365578)	Vascular endothelial growth factor A (human)	Complex **2**-(α-VEGF)	α-VEGF-photoCORM
Normal mouse IgG (sc-2025)	None	Complex **2**-(α-control)	α-Control-photoCORM

### Cell viability assays of Ab-photoCORMs in ovarian cancer cell model

**Scheme 1:**
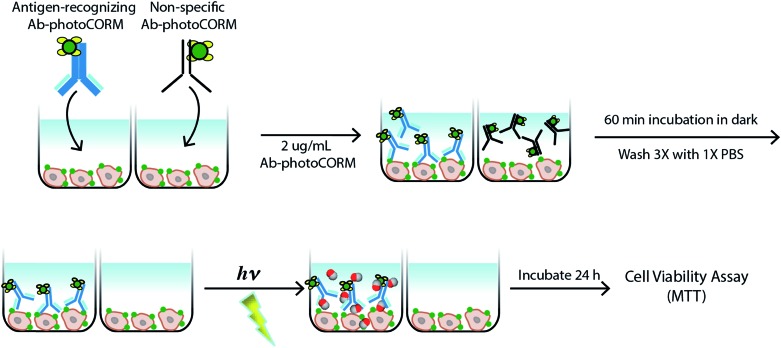
Live-cell, immunosorbent assay scheme utilized for assessment of the efficacy of antigen-recognition of an antibody-(photo-activated carbon monoxide-releasing molecule) conjugate (Ab-photoCORM) to deliver cytotoxic levels of carbon monoxide to ovarian cancer cells compared to a non-specific Ab-photoCORM conjugate.

The antigen-recognizing family of Ab-photoCORMs was finally assessed for their ability to localize and deliver cytotoxic levels of CO to OVCAR-5 and SKOV-3 cell cultures using a live-cell, immunosorbent assay (Scheme 1). Adherent cells were first treated with 2 µg mL^–1^ of Ab-photoCORMs for 60 min in the dark and then washed 3 times with 1x PBS to remove any non-specific association. Next fresh media was added to the cells and they were exposed to low-power visible light for 30 min for CO photorelease. After an incubation period of 24 h, cell viability was assessed by cellular reduction of MTT. The viability study clearly demonstrated that treatment of OVCAR-5 and SKOV-3 cells with Ab-photoCORM conjugates recognizing epitopes expressed in those ovarian cancer cell lines delivered cytotoxic levels of CO and dramatically decreased cell viability (Fig. 5B). α-Control-photoCORM did not significantly reduce cell viability (Fig. 5B), demonstrating that (a) CO alone was responsible for the cytotoxicity of the Ab-photoCORM complexes against the cancer cells, and (b) the presence of the right antigen on cancer cell surface was required for the targeted delivery of CO. Additionally, no significant cell death was observed either with light-inactivated Complex **1** or Complex **1** in the dark (Fig. S12†). Complex **2** by itself also did not exhibit significant toxicity to both ovarian cancer cells (Fig. S13†).

In order to establish a dose-dependence of Ab-photoCORM in CO-induced cell death, the α-HCAM-photoCORM was utilized in similar assay. As shown in Fig. 5C, α-HCAM-photoCORM elicited dose-dependent decreases in cell viability of the OVCAR-5 and SKOV-3 compared to α-Control-photoCORM. It is important to note that in previous experiments, similar photoCORMs with no conjugation with antibodies exhibited CO-induced cell death at much higher concentrations (10–50 µM range) compared to the present study where cell death is evident in presence of hundreds of picomoles of CO (Scheme S2†). While the greater potency of CO can be attributed to the improved localization of the Ab-photoCORM imparted by antigen recognition, much of this improved potency could likely be due to other mechanisms of action attributed ADCs generally, including antibody-mediated receptor signaling blockades and inflammatory responses due to the Fc component of the antibody.^21^ The relative contributions and synergism of these processes are poorly understood,^21^ but are nevertheless potent actions that can be attributed to the Ab-photoCORMs synthesized in this study. Taken together, these findings demonstrate the superior ability of the antigen-specific Ab-photoCORMs to accumulate onto ovarian cancer cells *via* recognition of surface proteins and deliver cytotoxic levels of CO in a much more efficient manner.

## Conclusions

The application of CO-releasing drugs face the challenge of strict site-specificity to avoid off-target effects of CO in normal cells. In the present work we have described an open strategy for site-specific and controlled delivery of CO to a desired biological target. Light-triggered release of a therapeutic molecule had, until this study, remained an unexplored approach for ADCs. This approach may be an effective strategy for reducing premature/off-target drug release by illumination of light directly to the tumor site. Furthermore, the frequency and length of illumination could be modulated to precisely control the kinetics of CO release from Ab-photoCORMs. In support of this strategy, a recent study has demonstrated the feasibility of light-triggered release of a CO-releasing material in an *in vivo* mouse models.^22^

The use of light for the remote control over the activity of pharmaceuticals, a concept known as photopharmacology, has a nearly 100 year old history in medicine and oncology.^23^ The successful clinical use of visible light to control drug activity in time and space to regulate biological processes is well documented.^24^–^26^ While photopharmacological treatment is naturally suitable for localized and exposed targets, optic fibers inserted through small and minimally invasive incisions allow for illumination of most body organs to be illuminated with intense, visible, broad spectrum light from non-laser sources.^27^

Photopharmacological approaches have even been successfully applied for metastatic ovarian cancer, where intraoperative and laparoscopic light sources were successfully used in photodynamic therapy, resulting in substantial benefits for patients in clinical trials.^28^ Visible light offers unparalleled therapeutic benefits as an external control element for pharmacological activity, which allows for the delivery and activity of photo-activatable pharmaceuticals with very high spatiotemporal precision. Furthermore, unlike chemicals, light exhibits high orthogonality towards biological systems with minimal contamination of the study subject and low to negligible toxicity.^23^ Visible light activation of photoCORMs and Ab-photoCORM conjugates for the therapeutic delivery of CO may soon mature beyond an academic strategy at this point in time considering the well-documented success of the clinical use of visible light in photopharmacology and photodynamic therapy.^23^,^24^

The high selectivity and diversity of monoclonal antibodies towards surface expressing antigens suggest that Ab-photoCORM conjugates could be designed to deliver CO to a wide range of cell/tissues with high specificity. Antibodies inherently exhibit a wide range of binding specificities due to amino acid residues contained within six short lengths, three each in the heavy and light chains of the antibody.^29^ As antibodies have the potential to recognize >10^12^ unique antigens,^30^ this can be exploited to improve the specificity of delivery of therapeutic molecules. The Ab-photoCORMs synthesized in this study have successfully exploited the antigen-recognition of antibodies to improve specificity of delivery of CO, a therapeutic, gaseous molecule. The Ab-photoCORMs reported here thus represent a novel class of ADCs that could be described as “immunoCORMs”.

The biotin-streptavidin linker utilized in these studies allow for the facile conjugation of the photoCORMs to any monoclonal antibody. Furthermore, the biotinylation of the photoCORM in this study was synthetically straightforward and performed under mild conditions. Biotinylation of not only other designed CORMs, but also hydrogen sulfide/nitric oxide donating molecules and nanoparticles is feasible. By this approach, the biotin-streptavidin linkage to monoclonal antibodies could be a new direction in the field of gasotransmitters, namely, the delivery of gaseous molecules driven by antibody-conjugation and antigen recognition.

Conventional ADCs require a number of specific properties in order to exhibit sufficient potency and stability. As one of the main mechanisms of drug resistance is ADC eflux,^31^ lack of susceptibility to multidrug resistant protein 1 (MDR1) is essential. CO, as a drug delivered by an ADC, could be intriguing in that it would be unaffected by efflux mechanisms of drug resistance like MDR1. Furthermore, traditional ADCs are limited by the frequency of internalization and trafficking through the endosomal-lysosomal pathway, a relatively infrequent event.^21^ The ability of CO to readily diffuse across cellular membranes could circumvent the need for antibody internalization *per se*. A photoCORM (a prodrug) conjugated to an antibody also requires that drug release is not dependent on linker cleavage or through complete degradation of the antibody within the tumor cell. The cleavable linkers impart small molecule drug ADCs with poor pharmacokinetics and circulation instability.^21^ The biotin-streptavidin linker used in this design is expected to maximize stability and mitigate the problems related to esterases and proteases within cellular milieu. The antibody-photoCORM conjugates (Ab-photoCORM) could be an intriguing tool for addressing some of the fundamental limitations of ADCs.

## Conflicts of interest

The authors declare no conflict of interest.

## Supplementary Material

Supplementary information

## References

[cit1] Motterlini R., Foresti R. (2017). Am. J. Physiol..

[cit2] Motterlini R., Otterbein L. E. (2010). Nat. Rev. Drug Discovery.

[cit3] Chakraborty I., Carrington S. J., Roseman G., Mascharak P. K. (2017). Inorg. Chem..

[cit4] Chakraborty I., Carrington S. J., Hauser J., Oliver S. R. J., Mascharak P. K. (2015). Chem. Mater..

[cit5] Chakraborty I., Jimenez J., Mascharak P. K. (2017). Chem. Commun..

[cit6] Schatzschneider U. (2015). Br. J. Pharmacol..

[cit7] Garcia-Gallego S., Bernardes G. J. L. (2014). Angew. Chem..

[cit8] Heinemann S. H., Hoshi T., Westerhausen M., Schiller A. (2014). Chem. Commun..

[cit9] Rimmer R. D., Pierri A. E., Ford P. C. (2012). Coord. Chem. Rev..

[cit10] Kawahara B., Moller T., Hu-Moore K., Carrington S., Faull K. F., Sen S., Mascharak P. K. (2017). J. Med. Chem..

[cit11] Kawahara B., Ramadoss S., Chaudhuri G., Janzen C., Sen S., Mascharak P. K. (2019). J. Inorg. Biochem..

[cit12] Beck A., Goetsch L., Dumontet C., Corvaia N. (2017). Nat. Rev. Drug Discovery.

[cit13] Pinto M. N., Chakraborty I., Sandoval C., Mascharak P. K. (2017). J. Controlled Release.

[cit14] Carrington S. J., Chakraborty I., Bernard J. M. L., Mascharak P. K. (2016). Inorg. Chem..

[cit15] Carrington S. J., Chakraborty I., Mascharak P. K. (2013). Chem. Commun..

[cit16] Hermanson G. T., (Strept)avidin-Biotin Systems, Bioconjug. Techniques, 3rd edn, 2013, pp. 465–505.

[cit17] Sacks J. D., Barbolina M. V. (2015). Biomolecules.

[cit18] Akhter M. Z., Sharawat S. K., Kumar V., Kochat V., Equbal Z., Ramakrishnan M., Kumar U., Mathur S., Kumar L., Mukhopadhyay A. (2018). Oncogene.

[cit19] Tsukioka M., Matsumoto Y., Noriyuki M., Yoshida C., Nobeyama H., Yoshida H., Yasui T., Sumi T., Honda K. I., Ishiko O. (2007). Oncol. Rep..

[cit20] Mukherjee S., Pal M., Mukhopadhyay S., Das I., Hazra R., Ghosh S., Mondal R. K., Bal R. (2017). J. Clin. Diagn. Res..

[cit21] Khera E., Thurber G. M. (2018). BioDrugs.

[cit22] Wang C. C., Li Y. Q., Shi X. Q., Zhou J. H., Zhou L., Wei S. H. (2018). Chem. Commun..

[cit23] Velema W. A., Szymanski W., Feringa B. L. (2014). J. Am. Chem. Soc..

[cit24] Hull K., Morstein J., Trauner D. (2018). Chem. Rev..

[cit25] Mehta Z. B., Johnston N. R., Nguyen-Tu M. S., Broichhagen J., Schultz P., Larner D. P., Leclerc I., Trauner D., Rutter G. A., Hodson D. J. (2017). Sci. Rep..

[cit26] Broichhagen J., Schonberger M., Cork S. C., Frank J. A., Marchetti P., Bugliani M., Shapiro A. M. J., Trapp S., Rutter G. A., Hodson D. J., Trauner D. (2014). Nat. Commun..

[cit27] Allison R. R., Cuenca R., Downie G. H., Randall M. E., Bagnato V. S., Sibata C. H. (2005). Photodiagn. Photodyn. Ther..

[cit28] Wierrani F., Fiedler D., Grin W., Henry M., Dienes E., Gharehbaghi K., Krammer B., Grunberger W. (1997). BJOG.

[cit29] Rose D. R. (1982). Am. J. Hematol..

[cit30] Alberts B.; Johnson A.; Lewis J., Martin R.; Roberts K.; and Walte P., The Generation of Antibody Diversity, Molecular Biology of the Cell, 4th edn, New York, Garland Science, 2002.

[cit31] Salomon P. L., Singh R. (2015). Mol. Pharm..

