# Supplementary material for: Diminished viability of human ovarian cancer cells by antigen-specific delivery of carbon monoxide with a family of photoactivatable antibody-photoCORM conjugates
Source: Chem Sci. 2019 Nov 20;11(2):467–73. doi: 10.1039/c9sc03166a (PMC7067254; doi:10.1039/c9sc03166a)
Supplement: Supplementary file 1 [file SC-011-C9SC03166A-s001.pdf]

Electronic Supplementary Material (ESI) for Chemical Science.
This journal is © The Royal Society of Chemistry 2019

Supporting Information

For

Diminished Viability of Human Ovarian Cancer Cells by Antigen-specific Delivery of Carbon Monoxide with a Family of Photoactivatable Antibody-photoCORM Conjugates

Brian Kawahara,† Lucy Gao,§ Whitaker Cohn,§ Julian P. Whitelegge,§ Suvajit Sen,‡ Carla Janzen,‡ Pradip K. Mascharak†*

Contribution from

†Department of Chemistry and Biochemistry, University of California, Santa Cruz, CA 95064, USA

§Pasarow Mass Spectrometry Laboratory, Jane and Terry Semel Institute for Neuroscience and Human Behavior, University of California at Los Angeles, Los Angeles, CA 90095, USA

and

‡Department of Obstetrics and Gynecology, David Geffen School of Medicine, University of California at Los Angeles, Los Angeles, CA 90095, USA

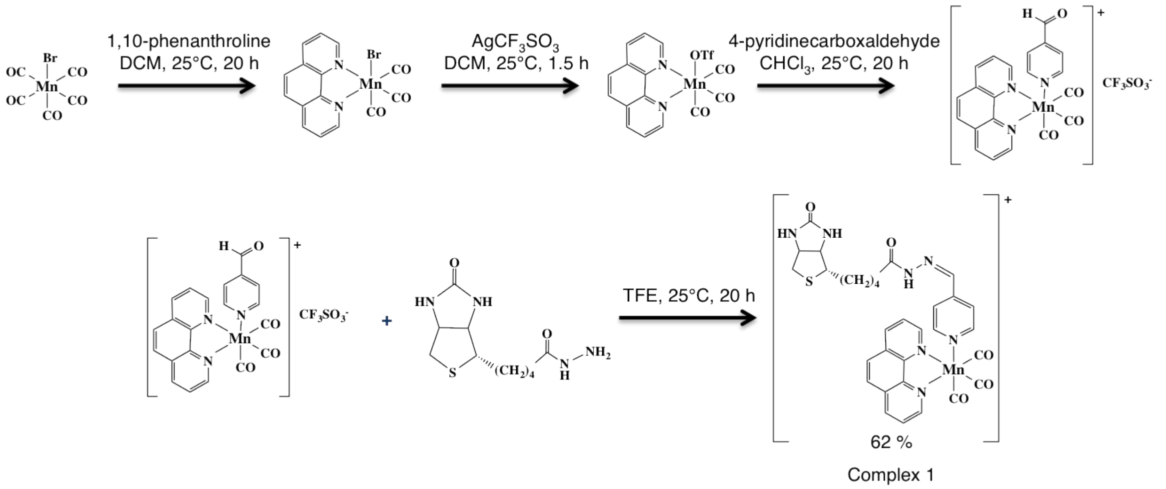

Scheme S1. Synthetic scheme for Complex **1**: biotinylated, photoactivatable CO-releasing molecule (photoCORM).

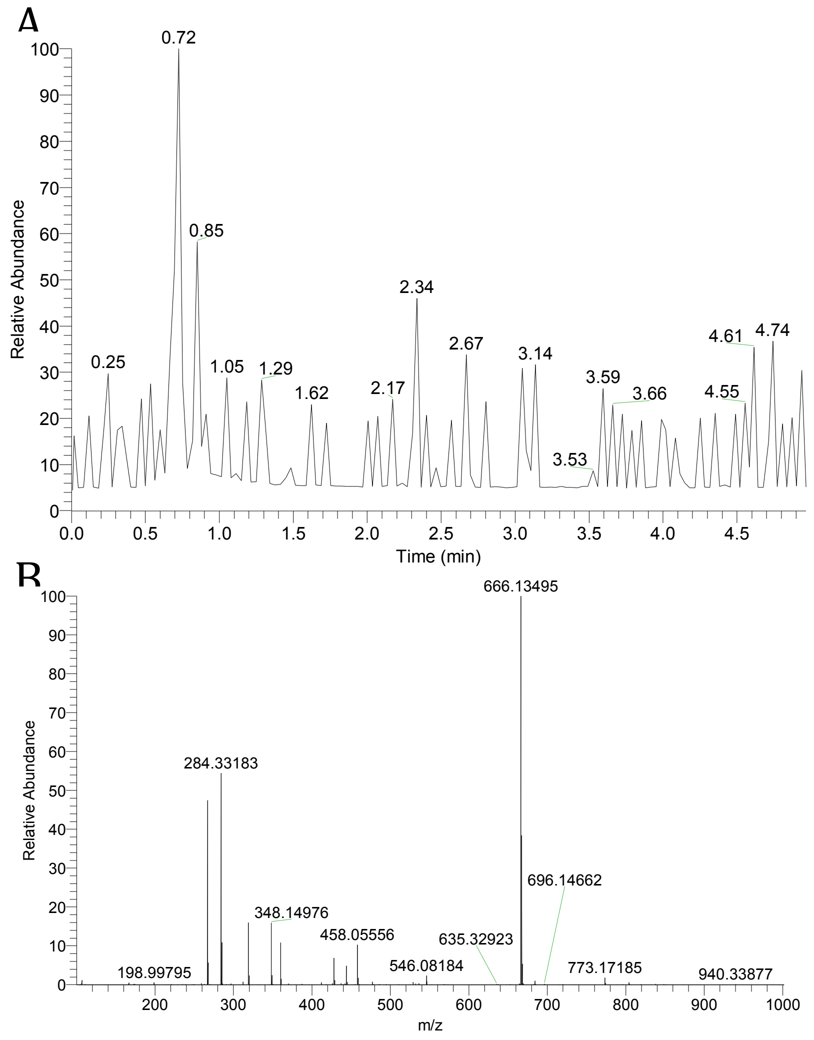

Figure S1. By electrospray ionization mass spectrometry (ESI-MS), (A) Total ion count (TIC) chromatogram of 0-5 min for a 5 min run of Complex **1** via flow injection analysis and (B) full mass spectrum (100-1000 m/z) for retention time=0.70-0.72. Found: 666.13495, calculated for $C_{31}H_{29}N_7O_5SMn$ 666.13314, Δ ppm = 3.4 ppm, Δ mDa = 2.2.

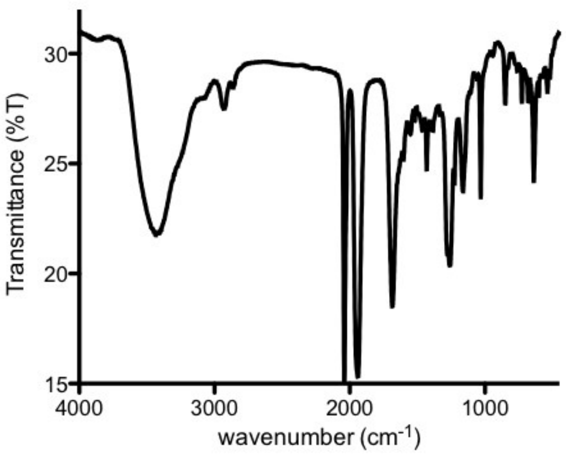

Figure S2. Infrared (IR) spectrum of Complex **1**. IR spectrum of solid Complex **1** was recorded in KBr matrix. $\nu_{C=O}$: (cm^{-1}): 2030, 1941, 1687.

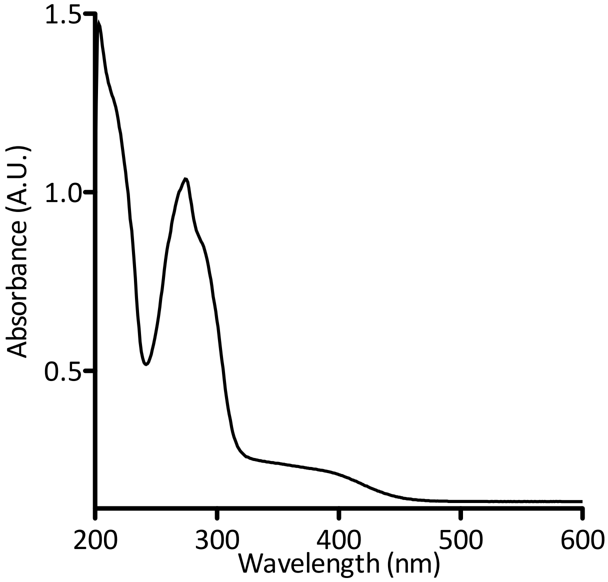

Figure S3. Electronic absorption spectrum of Complex **1** in 1x PBS, 25°C.

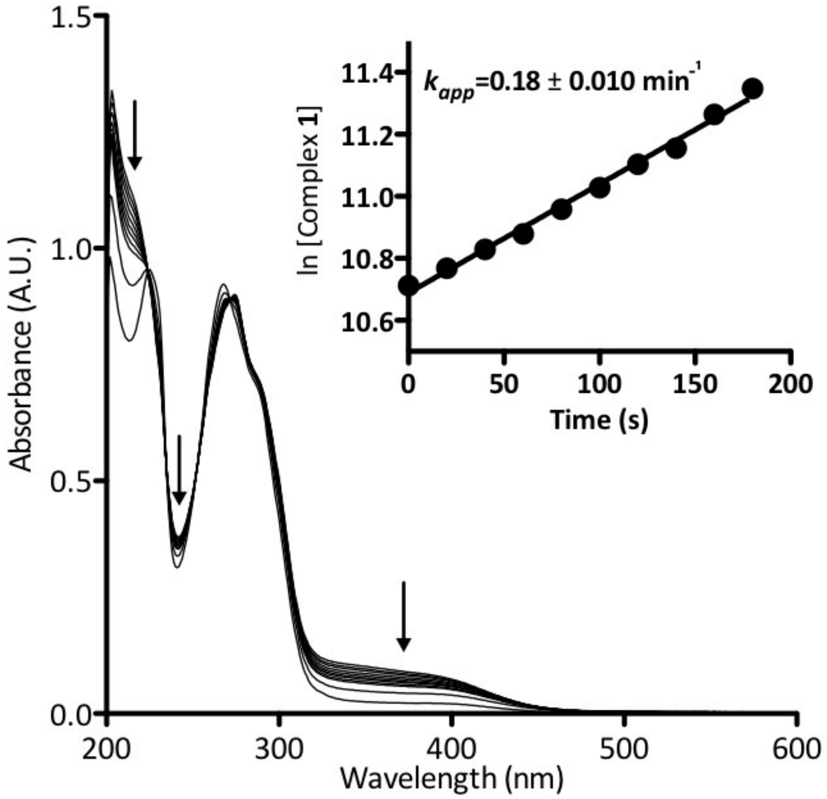

Figure S4. Electronic spectrum of Complex **1** upon illumination with low power (10 mW/cm^2), broadband visible light in 20-second intervals. Inset: Integration of the release rate for the photodegradation of Complex **1,** calculated at 390 nm, with low power (10 mW/cm^2), broadband visible light for indicated time.

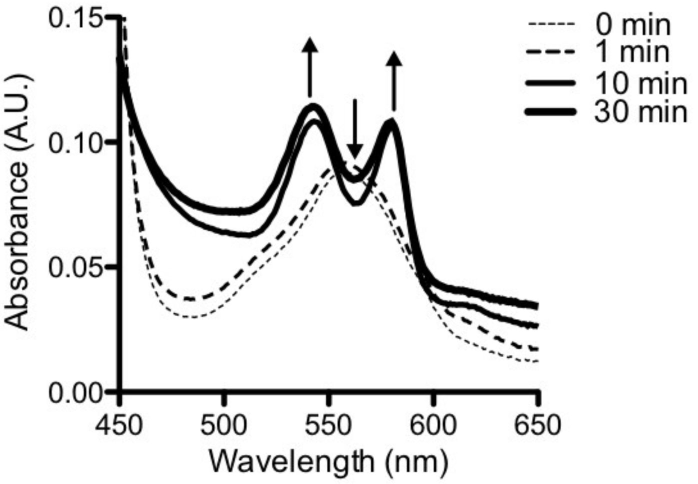

Figure S5. Myoglobin assay for CO release from Complex **1** dissolved and performed in 1X phosphate buffered saline (PBS), triggered by low power (10 mW/cm^2), visible light for indicated time.

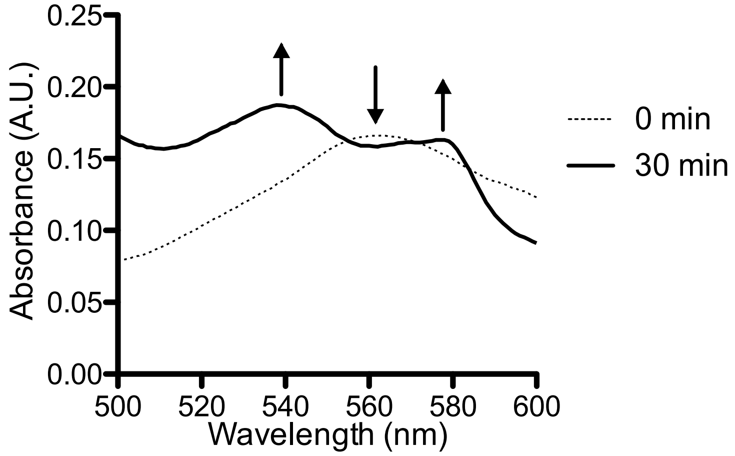

Figure S6. Myoglobin assay for CO release from Complex **1** dissolved in human serum and incubated for ≥1 h at 37°C, followed by exposure to low power (10 mW/cm^2), broadband visible light for 30 min.

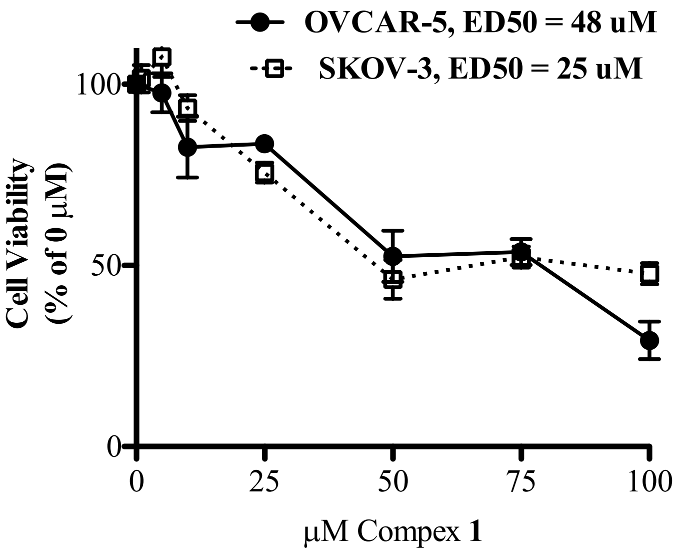

Figure S7. Effect of treatment of ovarian cancer cell lines with indicated concentrations of Complex **1** upon illumination with visible light on cell viability, measured 24 h post-treatment in ovarian cancer cell lines OVCAR-5 and SKOV-3. Data representative of n=3 independent experiments.

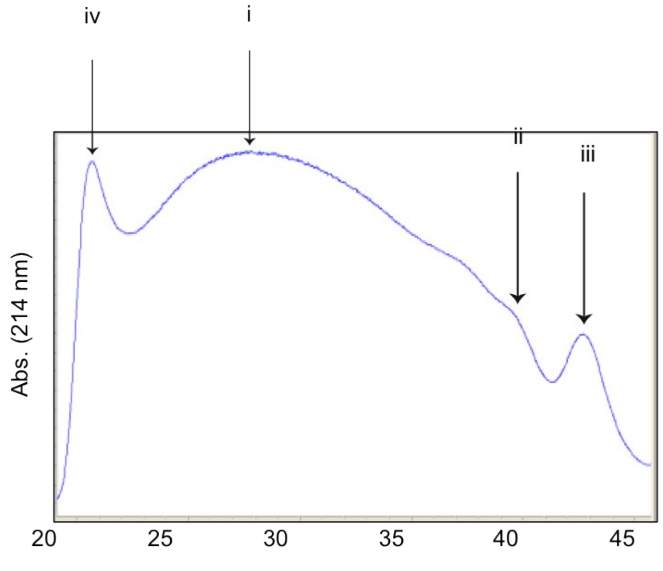

Figure S8. Chromatogram of Complex **2** following size exclusion chromatography. [retention time, ~molecular weight, identity] (i) [28.4 min, 366 kDa, IgG + 4 streptavidin]. (ii) [39.6 min, ~155 kDa, IgG + 0 streptavidin]. (iii) [42.7 min, ~121 kDa, IgG Fragments]. (iv) [20.6 min, ~659 kDa, void volume].

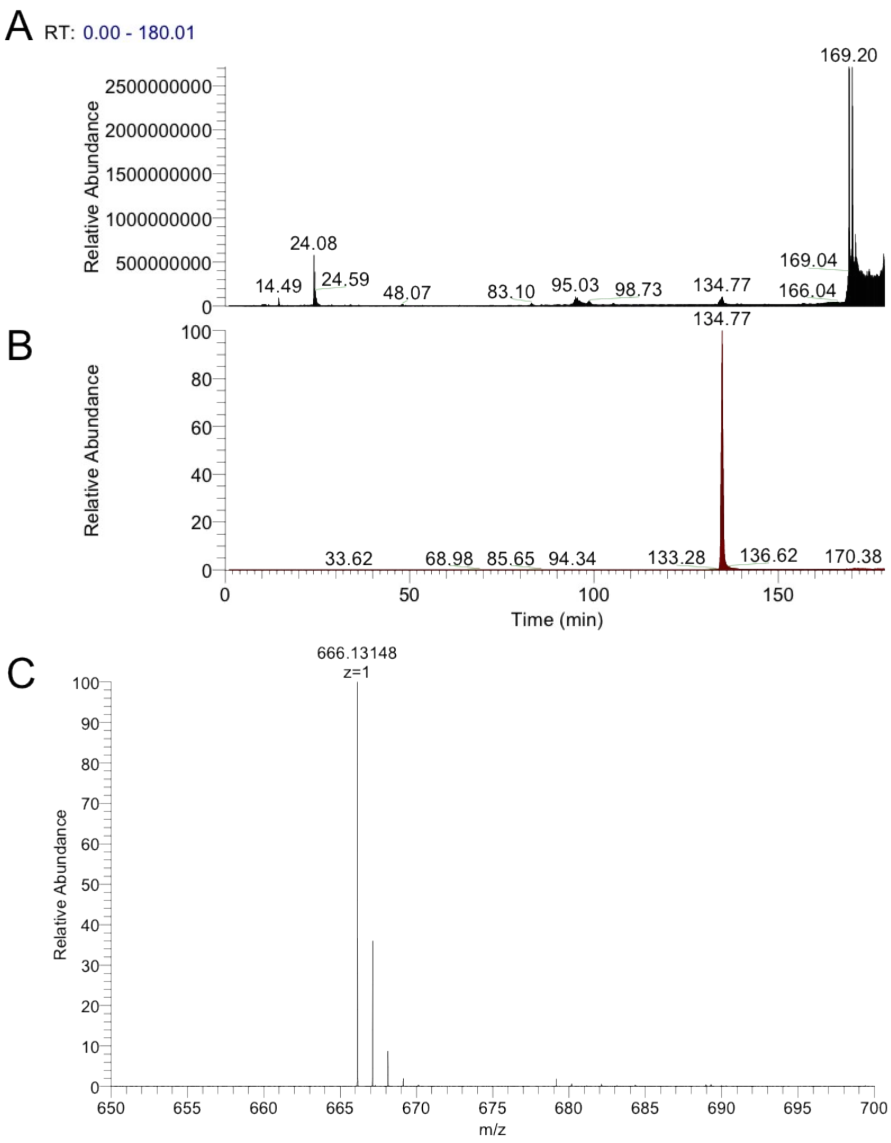

Figure S9. Detection of Complex **1** in tryptic digest of antibody-photoCORM conjugate (Ab-photoCORM). (A) Total ion count (TIC) of Ab-photoCORM sample. (B) Chromatogram of Ab-photoCORM, mass filter range m/z = 666.12593-666.13925. (C) Full mass spectrum at retention time 134.16-135.34 min.

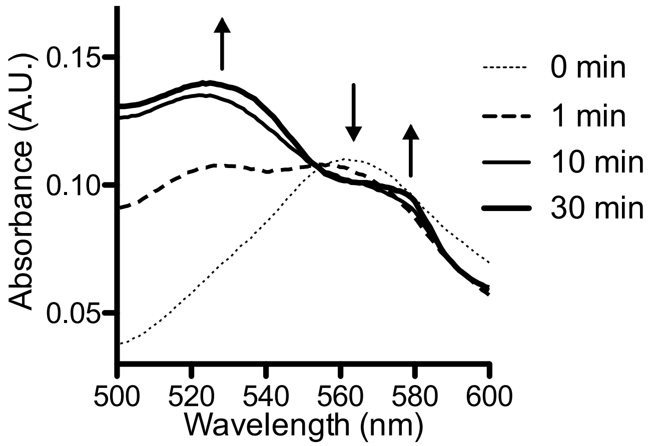

Figure S10. Myoglobin assay for CO release from antibody-photoCORM conjugate (Ab-photoCORM) dissolved in 1X phosphate buffered saline (PBS), triggered by low power (10 mW/cm^2), broadband visible light for indicated times.

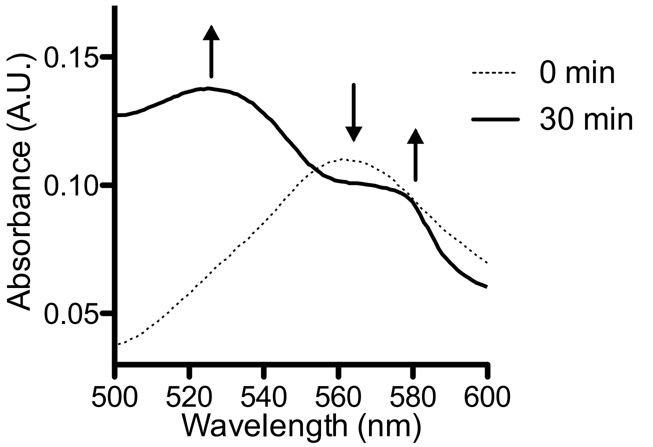

Figure S11. Myoglobin assay for CO release from the antibody-photoCORM conjugate (Ab-photoCORM) after ≥1 h incubation in human serum at 37°C, followed by exposure to low power (10 mW/cm^2), broadband visible light for 30 min.

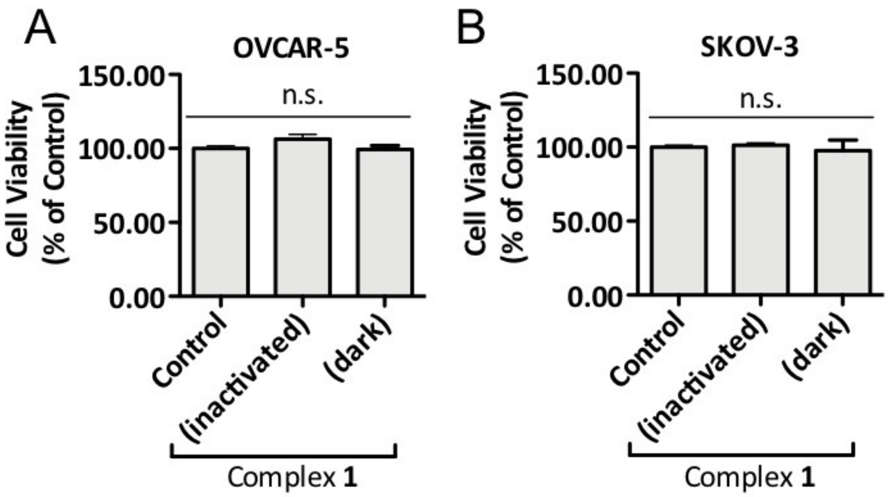

Figure S12. Cell viability of ovarian cancer cell lines OVCAR-5 and SKOV-3 treated with light-inactivated Complex **1** and Complex **1** in the dark to assess the cytotoxicity of non-CO components of Complex **1**. Data representative of n=3 independent experiments. (* $p<0.05$)

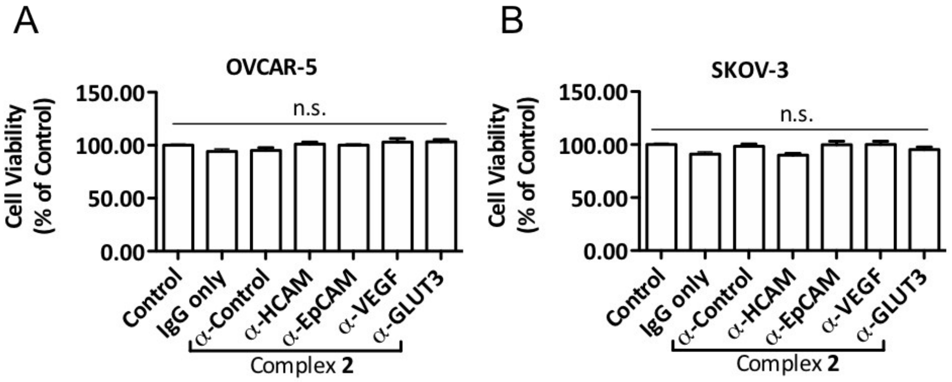

Figure S13. Cell viability, as measured by reduction of MTT 24 h post-treatment, of (A) OVCAR-5 and (B) SKOV-3 treated with 2 μg/mL Complex **2** and control antibodies. Family of Complex **2** constructed from mouse monoclonal antibodies raised against human HCAM (α-HCAM), EpCAM (α-EpCAM), GLUT3 (α-GLUT3) and VEGF-1 (α-VEGF). Contol treatments with vehicle control (Control), IgG without streptavidin (IgG) and Complex **2** synthesized from control mouse IgG (α-Control) included. Data representative of n=3 independent experiments. (* $p<0.05$)

$$\left(\frac{\text{2-10 µg Ab-photoCORM}}{\text{mL}}\right)\left(\begin{matrix}\text{0.2 mL cell}\\ \text{culture media}\end{matrix}\right)\left(\frac{\text{1-4 mol streptavidin}}{\text{1 mol Ab-photoCORM}}\right)\left(\frac{\text{4 mol Complex }\mathbf{1}}{\text{1 streptavidin}}\right)\left(\frac{\text{3 mol CO}}{\text{1 mol Complex }\mathbf{1}}\right)= \begin{matrix}\text{~23-262 pmol}\\ \text{CO}\end{matrix}$$

Scheme S2. Calculations for estimation of molar release of CO from antibody-photoCORM conjugates.

Experimental Section

Materials

Biotin-hydrazide (A8007-100mg) was procured from Apex Biotech, Ltd. (Xuzhuang, Shaanxi, PRC). Mouse monoclonal antibodies raised against HCAM (sc-7297), EpCAM (sc-53277), GLUT3 (sc-74399), VEGF-1 (365578) and normal mosue IgG (sc-2025) were obtained from Santa Cruz Biotechnology (Santa Cruz, CA, USA). All other chemicals were purchased from Sigma-Aldrich (St. Louis, MO, USA) unless otherwise stated.

Synthesis of biotin-photoCORM (Complex 1)

$[Mn(CO)_5(Br)]$ (100 mg, 0.36 mmol) and 1,10-phenanthroline (phen) (66 mg, 0.36 mmol) was dissolved in 25 mL dichloromethane (DCM) and allowed to stir in the dark for 20 h at 25°C. The solution, initially dark yellow, was dried down under vacuum to a yellow powder. Next, 1.5-fold excess $AgCF_3SO_3$ (140 mg, 0.54 mmol) was dissolved in 20 mL DCM and added to the yellow product, and allowed to stir for 1.5 h at 25°C in the dark. The cloudy green solution was subsequently filtered through a wet Celite pad, and the filtrate was evaporated to dryness. The resulting yellow powder was dissolved in 50 mL chloroform. To that stirring solution, 4-pyridinecarboxaldehyde (pyAl) (385 mg, 3.6 mmol) was added drop wise and allowed to stir for 20 h at 25°C in the dark. The next day, the solution was dried down under vacuum, revealing orange/yellow microcrystals of $[Mn(CO)_3(phen)(PyrAl]CF_3SO_3$ (155 mg, 0.27 mmol, 75%). To 223 mg (0.39 mmol) of $[Mn(CO)_3(phen)(PyrAl]CF_3SO_3$, biotin-hydrazide (100 mg, 0.39 mmol) dissolved in 20 mL of freshly distilled 2,2,2-trifluoroethanol was added, in a similar manner to a previous study.[1] The dark yellow solution was allowed to stir for 20h at 25°C in the dark. The yellow brown solution was concentrated under vacuum to ~2 mL, then chromatographed on a basic alumina column (50-200 µm particle diameter). The column was then washed with DCM (to remove unreacted $[Mn(CO)_3(phen)(PyrAl]CF_3SO_3$ and biotin-hydrazide and finally Complex **1** was eluted with DCM/methanol (3/2 v/v).

^1H NMR (400 MHz, $[D_4]$-methanol]): δ=1.37-1.84 (m, 6H), 2.30 (t, 2H, 7.4 Hz), 2.68 (m, 1H), 2.89 (m, 1H), 3.18 (m, 1H), 4,24 (m, 1H), 4.47 (m, 1H), 7.53 (m, 2H), 7.86 (s, 1H), 8.20 (m, 4H), 8.38 (m, 2H), 8.87 (m, 2H), 9.88 (m, 2H); IR (KBr): v = 2039, 1939, 1685 cm^{-1}(C=O); HRMS (ESI): *m/z* calcd for $C_{31}H_{29}N_7O_5SMn$: 666.13315 [*M*+]; found: 666.13539, Δ ppm = 3.4 ppm, Δ mDa = 2.2; elemental analysis calcd (%) for $C_{31}H_{29}N_7O_5SMn$: C 55.86, H 4.35, N 14.71,

O 12.01, S 4.80, Mn 8.26; found: C 55.84, H 4.39, N 14.71, O 12.01, S 4.80, Mn 8.25.

Physical Measurements

^{1}H NMR spectra of Complex **1** were collected at 298 K on a Varian Unity Inova 500 MHz instrument. FT-IR of Complex **1** was collected on a PerkinElmer Spectrum-One FT-IR. UV–vis data of Complex **1** were recorded on a Varian Cary 50 UV–vis spectrophotometer.

Myoglobin Assay

Horse heart myoglobin was dissolved in 1X PBS, pH=7.4 to a final concentration of 50 μM and reduced with 0.1 % sodium dithionite in quartz cuvette under aerobic conditions. In a second cuvette, Complex **1** was dissolved in 1X PBS to a final concentration of 50 μM. Antibody-photoCORM conjugates (Ab-photoCORM) were dissolved in 1X PBS to a final concentration of 10 μg/mL. For myoglobin assays performed in human serum, either Complex **1** or Ab-photoCORMs was dissolved to final concentrations of 50 μM or 10 μg/mL respectively in human serum, followed by ≥1h incubation at 37°C. Photogenerated CO, triggered by low power, broadband visible light (10mW/cm^2) from Complex **1** or Ab-photoCORM was released into the headspace and transferred to the reduced Mb solution via a cannula and positive pressure with N_2(g). The extent of the conversion of Mb to carboxymyoglobin (MbCO) was monitored by the change in absorbance at 540 nm, a reliable determination of CO-release from organometallic carbonyl complexes.[2] The source of low power, broadband visible light was an IL 410 Illumination System purchased from Electro Optical Components, Inc. (Santa Rosa, CA, USA). Visible light power was measured with a Field MaxII-TO laser power meter purchased from Coherent (Palo Alto, CA, USA).

Photolysis Experiments

The rate of CO release (k_{CO}) for Complex **1** at 25 °C in 1x PBS was assessed with in 1 cm x 1 cm quartz cuvettes. The k_{CO} of Complex **1** (concentration = 3.0 × 10^{-5} M, 390 nm, 25°C) was determined by recording the electronic absorption spectra, monitoring changes in the spectra following exposure to light at regular intervals. k_{CO} was then calculated from the ln[Complex **1**] versus time (*t*) plot.

Synthetic strategy of streptavidin-conjugated mouse IgG (Complex 2)

Conjugation of 1 mg mouse IgG, either control or antigen-specific IgG, with streptavidin was performed utilizing the Streptavidin Conjugation Kit (ab102921, Cambridge, MA, USA). Native gel electrophoresis and size exclusion chromatography were used to analyze and characterize streptavidin-conjugated antibodies. Complex **2** was quantified for use in subsequent cellular studies by measuring total protein using a Pierce™ BCA Protein Assay Kit (23225, ThermoFisher Scientific, Waltham, MA).

Native Gel Electrophoresis

2 μg of streptavidin-IgG conjugate was combined with native loading dye (62.5 mM Tris-HCl, pH=7.4, 40% glycerol and 0.01% bromophenol blue) and loaded onto a 4-12% Mini-PROTEAN TGX Precast Protein Gels (#4561095, Bio-Rad, Hercules, CA, USA) and separated under non-reducing, native conditions. Protein bands were visualized using Coomassie Brilliant Blue R-250 (#161-0436).

Size Exclusion Chromatography

Separation and simultaneous UV absorbance detection at 214 nm of streptavidin-conjugated antibodies and antibody-photoCORM conjugates was performed using a 7.5 D x 60 cm, 3 μm Tosoh TSK G4000SW (stainless steel) column. The column was preconditioned with molecular weight standards. The mobile phase was prepared with 137 mM NaCl, 2.7 mM KCl, 4.3 mM Na_2HPO_4, 1.47 mM KH_2PO_4, pH = 6.8 and sterile filtered and degassed prior to use. Separation species based on size was performed at a flow rate of 0.5 mL/min, 25 °C. Fractions of streptavidin-conjugated antibodies and antibody-photoCORM conjugates were further characterized by bottom up proteomics.

Synthesis of antibody-photoCORM Conjugates (Ab-photoCORM)

Complex **2** (100 μg, ~273 pmol) pre-dissolved in 500 μL 1X PBS was reacted with excess Complex **1** (40.0 ng, 60 nmol) pre-dissolved in 500 μL 1X PBS for 1h at 25°C in the dark. The antibody-photoCORM conjugates (Ab-photoCORMs) were purified using size exclusion chromatography. Bottom up proteomics and HPLC-MS/MS analysis was utilized to characterize the composition of Ab-photoCORMs. Detection of Complex **1** in Ab-photoCORMs was observed in full MS scans in the bottom up proteomic assays.

Bottom Up Proteomics Analysis

10 μg of each antibody-photoCORM conjugate, as determined by BCA Protein Assay, were solubilized in 200 μL lysis buffer (12 mM sodium lauroyl sarcosine, 0.5% sodium deoxycholate, 50 mM triethylammonium bicarbonate (TEAB)) followed by 10 min bath sonication and heating at 95°C for 5 min. The samples were then diluted to 0.5 mg total protein/mL with lysis buffer, then a 100 μL aliquot was treated with 5 mM tris(2-carboxyethyl) phosphine (TCEP) prepared in 50 mM aqueous TEAB at 60°C for 30 min. Next, the samples were treated with 10mM chloroacetamide, prepared in 50 mM TEAB, for 30 min at 25°C in the dark. Samples were diluted 5-fold in 50 mM TEAB, then incubated overnight with Sequencing Grade Modified Trypsin (1:100, g trypsin: g total protein). The next day, an equal volume of ethyl acetate/trifluoroacetic acid(TFA, 100/1, v/v) was added to samples, followed by 5 min vigorous vortexing and centrifugation (13,000 x g, 5 min). Desalting of samples was performed similar to that previously described.[3] Dried samples were reconstituted in acetonitrile/water/TFA (2/98/0.1, v/v/v), loaded onto a C18-silica disk (3M, Maplewood, MN, USA) placed inside a 200 μL pipet tip. Prior to sample loading onto the disk, it was equilibrated with methanol (20 μL), acetonitrile/water/TFA (20 μ L, 80/20/0.1, v/v/v), then finally acetonitrile/water/TFA (2/98/0.1, v/v/v). The

samples loaded onto the disks were washed with acetonitrile/water/TFA (20 µL, 2/98/0.1, v/v/v) and eluted with acetonitrile/water/TFA (40 µL, 80/20/0.1, v/v/v). Eluents were concentrated under vacuum centrifugation and reconstituted in 10 µL water/acetonitrile/formic acid, 98/2/0.1, v/v/v). 5 µL aliquots were injected onto a reverse phase nanobore HPLC column (AcuTech Sceintiic, C18, 1.8 µm particle size, 360 µm x 20 cm, 150 µm ID), equilibrated in water/acetonitrile/formic acid (98/2/0.1, v/v/v: min/%; 0/0, 5/3, 18/7, 74/12, 144/24, 153/27, 162/40, 164/80, 174/80, 176/0, 180/0) using an Eksigent NanoLC-2D system (Sciex, Framingham, MA, USA). The flow from the column was directed towards nanospray ionization source connected to a Q Exactive™ Hybrid Quadrupole-Orbitrap™ Mass Spectrometer (Thermo Fisher Scientific). Data-dependent mass spectra were acquired alternating between full scan (m/z 350-2000, automated gain control target 3 x 10^3, 50 ms maximum injection time, FWHM resolution 70,000 at m/z 200) and up to 10 MS/MS scans (quadrupole isolation of charge states ≥1, isolation width 1.2 Th) with optimized fragmentation conditions (normalized collision energy of 32, dynamic exclusion of 30 s, AGC target 1 x 10^6, 100 ms maximum injection time, FWHM resolution 35,000 at *m/z* 200). Analysis of raw data and peptide/protein identification of the antibody-photoCORM conjugates was performed using Mascot to search the UniProt-Mouse database. Common Contaminants database was also searched to identify streptavidin. Probability based scoring was used to determine significance of data, where reported scores = -10 x $Log_{10}(P)$, where P is the absolute probability that the observed match between the experimental data and the database sequence is a random event.[4,5] Scores >67 are considered significant ($p<0.05$).[4,5] Complex **1** associated with Ab-photoCORM was observed in the full MS scan data (Figure S6).

Cell culture

Ovarian cancer cell lines OVCAR-5 and SKOV-3 were obtained from American Type Culture Collection (Manassas, VA, USA). OVCAR-5 and SKOV-3 were grown in RPMI 1640 Medium (11875119, ThermoFisher Scientific) supplemented with 10% fetal bovine serum (FBS, 16000) and 100 U/mL penicillin-streptomycin (15070063) were all purchased from ThermoFisher Scientific. Cells were passaged ≤ 10 times after acquisition from the manufacturer.

Cell Viability (MTT) Assay

Cell viability was assessed by the cellular reduction of tetrazolium dye MTT performed in 96-well tissue culture plates. 2 × 10^3 cells/well were allowed to seed overnight in a 37 °C incubator + 5% CO_2. The following day, cells were treated as indicated with Complex **1** or Complex **2**, then assessed for viability 24 h post-treatment. Following removal of cell culture media, 0.5 mg/mL MTT dissolved in fresh 1× DMEM was added and allowed to incubate for 2 h in a 37 °C incubator + 5% CO_2. Cell viability was quantified by measuring the relative amount of MTT reduced to insoluble formazan. Following solubalization of formazan in 10% SDS

+ 0.01 N HCl, formazan was measured by taking the absorbance at 570 mm, reference wavelength taken at 690 nm.

Western Analysis

Whole cell lysates were extracted using RIPA lysis buffer (150 mM NaCl, 5 mM EDTA pH 8.0, 50 mM Tris pH 8.0, 1% Triton X-100, 0.5% sodium deoxycholate, 1% SDS and 1× protease inhibitor cocktail. BCA Protein Assay assayed soluble fractions for total protein content. 20 µg of soluble cell lysates from samples were resolved on 10% SDS-PAGE gel and transferred to poly(vinylidene difluoride) (PVDF) membrane. All following blocking and antibody solutions were prepared in 1x PBS + 0.1% Tween 20. Membranes, following blocking in 5% nonfat dried milk for 18 h at 4°C, were probed with primary (1:1000 dilution) antibody overnight at 4°C and then horseradish peroxidase (HRP)-conjugated secondary (1:10,000 dilution) antibody for 1 h at 25°C. Immunofluorescent signals were amplified with Pierce ECL Plus Western blotting substrate (32132, ThermoFisher Scientific).

Live-cell Immunosorbent Assay

2 x 10^3 cells/well of 96-well tissue culture plates were allowed to seed overnight at 37°C + 5% CO_2. The next day, cells were treated as indicated with 0-10 µg/mL antibody-photoCORM conjugates (Ab-photoCORM), as measured by BCA Protein Assay. Immunosorbence of Ab-photoCORMs to the adherent live cells was allowed to occur for 60 min in the dark at 37°C + 5% CO_2. α-Control Ab-photoCORM, utilizing control mouse IgG (sc-2025, Santa Cruz Biotechnology), was utilized as control to assess the specificity of the other antibody-photoCORM conjugates. Following incubation, the media was gently aspirated, followed by three 250 µL washes with 1X PBS in the dark to remove any non-specific binding. 100 µL fresh cell culture media was added, followed by illumination with low power, visible light for 30 min to trigger release of CO from any Ab-photoCORM present after immunosorbence and washing. Cell viability, as measured by the reduction of MTT, was assayed 24 h post-illumination of light.

Statistical Analysis

Data are expressed as the mean ± standard error mean (range) or as percentage of control value where indicated. Comparisons between two groups were made using the Student’s t-test. Comparisons between more than two groups were made using the One-way ANOVA/ Tukey's post hoc test. p-values < 0.05 were considered statistically significant. All calculations were performed using GraphPad Prism software package (GraphPad Software Inc., San Diego, USA).

Supporting References

[1] M. Salmain, N. Fischer-Durand, L. Cavalier, B. Rudolf, J. Zakrzewski, G. Jaouen, *Bioconjug. Chem*. **2002**, *13*, 693-698.

[2] A. J. Atkin, J. M. Lynam, B. E. Moulton, P. Sawle, R. Motterlini, N. M. Boyle, M. T. Pryce, I. J. S. Fairlamb, *Dalton Trans.* **2011**, *40*, 5755-5761.

[3] J. Rappsilber, M. Mann, Y. Ishihama, *Nat. Protoc.* **2007**, *2*, 1896-1906.

[4] T. Koenig, B. H. Menze, M. Kirchner, F. Monigatti, K. C. Parker, T. Patterson, J. J. Steen, F. A. Hamprecht, H. Steen, *J. Proteome Res.* **2008**, *7*, 3708-3717.

[5] D. J. C. Pappin, P. Hojrup, A. J. Bleasby, *Curr. Biol.* **1993**, *3*, 327-332.
